# Prominin-1 Modulates Rho/ROCK-Mediated Membrane Morphology and Calcium-Dependent Intracellular Chloride Flux

**DOI:** 10.1038/s41598-019-52040-9

**Published:** 2019-11-04

**Authors:** Akiko Hori, Kenji Nishide, Yuki Yasukuni, Kei Haga, Wataru Kakuta, Yasuyuki Ishikawa, Matthew J. Hayes, Shin-ichi Ohnuma, Hiroshi Kiyonari, Kazuhiro Kimura, Toru Kondo, Noriaki Sasai

**Affiliations:** 1 Developmental Biomedical Science, Division of Biological Sciences, Nara Institute of Science and Technology, 8916-5, Takayama-cho, Ikoma 630-0192 Japan; 2 Laboratory for Cell Lineage Modulation, Center for Developmental Biology, 2-2-3, Minatojima-Minamimachi, Chuo-ku, Kobe 650-0047 Japan; 3 Department of Systems Life Engineering, Maebashi Institute of Technology, 460-1, Kamisadori-cho, Maebashi, Gunma 371-0816 Japan; 4 Faculty of Brain Sciences, UCL Institute of Ophthalmology, University College London, 11-43 Bath Street, London, EC1V 9EL United Kingdom; 5 Laboratory for Animal Resources and Genetic Engineering, RIKEN Center for Biosystems Dynamics Research, 2-2-3, Minatojima-Minamimachi, Chuo-ku, Kobe 650-0047 Japan; 6 Department of Ophthalmology, Yamaguchi University School of Medicine, 1-1-1 Minamikogushi, Ube, 755-0046 Japan; 7 Division of Stem Cell Biology, Institute for Genetic Medicine, Hokkaido University, Kita-15, Nishi-7, Kita-Ku, Sapporo 060-0815 Japan

**Keywords:** Mechanisms of disease, Ion channel signalling

## Abstract

Membrane morphology is an important structural determinant as it reflects cellular functions. The pentaspan membrane protein Prominin-1 (Prom1/CD133) is known to be localised to protrusions and plays a pivotal role in migration and the determination of cellular morphology; however, the underlying mechanism of its action have been elusive. Here, we performed molecular characterisation of Prom1, focussing primarily on its effects on cell morphology. Overexpression of Prom1 in RPE-1 cells triggers multiple, long, cholesterol-enriched fibres, independently of actin and microtubule polymerisation. A five amino acid stretch located at the carboxyl cytosolic region is essential for fibre formation. The small GTPase Rho and its downstream Rho-associated coiled-coil-containing protein kinase (ROCK) are also essential for this process, and active Rho colocalises with Prom1 at the site of initialisation of fibre formation. In mouse embryonic fibroblast (MEF) cells we show that Prom1 is required for chloride ion efflux induced by calcium ion uptake, and demonstrate that fibre formation is closely associated with chloride efflux activity. Collectively, these findings suggest that Prom1 affects cell morphology and contributes to chloride conductance.

## Introduction

Each cell has a unique shape corresponding to its specific functions. Cell morphology is mainly controlled by the combination of cytoskeletal proteins and the dynamics of plasma membrane curvature and invagination.

Cell protrusions include structures such as cilia, cytonemes, microvilli, and retraction fibres^1,2^. Cilia contain microtubules, whereas cytonemes and retraction fibres contain actin, and they act as antennae for the extracellular stimuli and/or as the scaffold for the cell movement^1–3^. Microvilli are also membrane protrusions rich in cholesterol^4^, and act to increase cell surface area, often at interfaces with the environment, to aid in efficient incorporation of extracellular materials into the body. Because the protrusions closely correlate with many cell functions, the mechanisms of cell shape regulation are among the central questions of cell biology.

In the vertebrate retina, the photoreceptor cell has a long cell shape, and is divided into different functional compartments. Specialised membrane protrusions contribute to all elements of retinal structure; the outer segment, for instance, comprises the discs that are responsible for light perception^5^. In addition, the retinal pigmented epithelium (RPE) cells, localised at the back of the photoreceptor cells, also have a unique shape with long protrusions and are assumed to play roles in the photoreceptor disc metabolism^6^.

Prominin-1 (CD133, Prom1) encodes a pentaspan transmembrane glycoprotein, highly expressed in the retina, as well as in the kidney and testis^7^. In the retina, Prom1 is expressed in photoreceptor cells^8^ and in RPE cells^6,9^, and is recognised as a crucial gene in the retinal homeostasis^8^. In pedigrees with mutations in the Prom1 gene, individuals carrying the homologous mutation suffer from inherited macular dystrophies termed as Stargardt’s disease and retinitis pigmentosa (RP); the symptoms begin in childhood, followed by gradual vision loss^10–12^. In previous studies using Prom1 gene deficient mice (*Prom1*KO), progressive retinal degeneration in the postnatal stages has been demonstrated^10,12–14^. In *Prom1*KO mice, photoreceptor development and retinal structure at the perinatal stages are normal, but the membrane structure of the photoreceptor cells starts deforming once the eyes open^14^. As Prom1 has been shown to be localised at the protrusive structures in a number of cellular contexts^15^, it is reasonable to predict that Prom1 would be involved in the retinal function through regulation of the morphogenesis of the retinal cells. However, as the knowledge of the molecular characteristics and the signalling pathway(s) induced by Prom1 is still fragmented, the mechanisms by which the retinal degeneration initiates remain unclear.

It has been demonstrated that two carboxyl cytoplasmic tyrosines of Prom1 protein are phosphorylated by the oncogenic protein kinases Fyn and Src^16^. Moreover, phosphorylated Prom1 interacts with PI3K (phosphoinositide 3-kinase) and activates AKT, which is involved in self-renewal and tumorigenicity of the glioma stem cells^17^. However, whether this activation module and the signalling pathways are active in different contexts is elusive. Importantly, in photoreceptor cells, PI(3,4,5)P3, the product of PI3K, is predominantly localised at the inner segment, whereas Prom1 is mainly localised at the outer segment^8,18^. The deletion of p85α, a subunit of PI3K, does not lead to a severe retinal degeneration^19^. This suggests that the essential signalling pathway in maintenance of the retina functions is distinct from the one mediated by PI3K.

In this study, we attempted molecular characterisation of the Prom1 protein, and identify a signalling pathway triggered by Prom1. We found that cell morphology was considerably altered by the overexpression of Prom1 in a retinal pigmented epithelium derived cell line; numerous long membrane fibres, enriched in cholesterol, were formed. By using this as the evaluation criterion, we identified the essential amino acids and the downstream signalling pathway which trigger this morphological change. Importantly, chloride efflux is closely associated with the formation of fibres on the cell membrane. We discuss the involvement of Prom1 in membrane morphogenesis through the activity of chloride conductance.

## Results

### Overexpression of Prom1 induces the formation of the fibral structure at the rear of the moving human RPE1 cells

**Figure 1:**
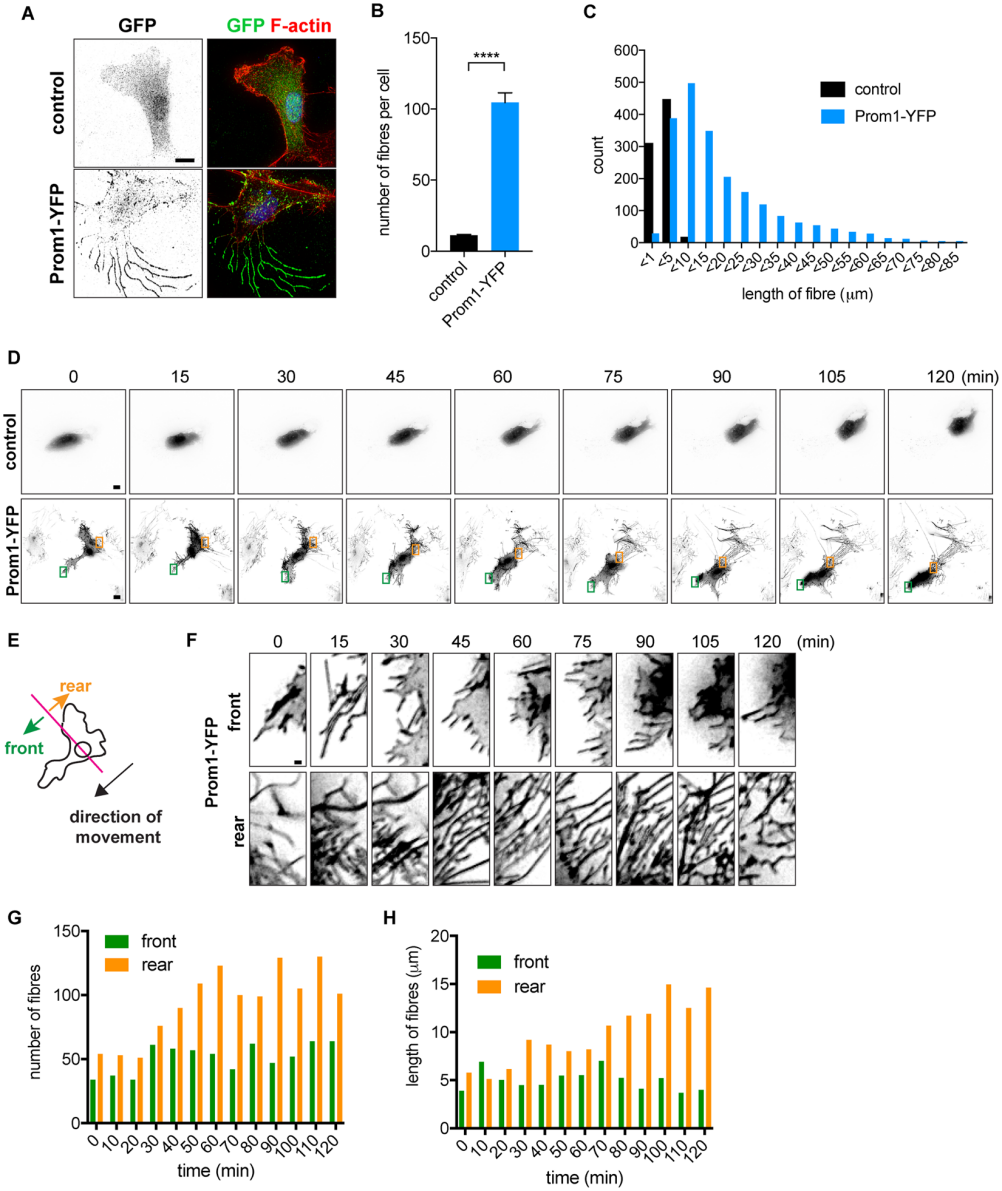
Prom1 induces cell membrane extensions. (**A**) Prom1-YFP induces fibre formation. Expression plasmids conveying control *YFP* or *Prom1-YFP* were transfected into the RPE1 cells and were harvested for 24 hours after the transfection. Cells were stained with GFP antibody (green) or phalloidin (red). (**B**,**C**) Quantitative data for the numbers (**B**) and lengths (**C**) of the fibres. In (**B**), 20 cells were analysed in each experiment, and the experiments were repeated four times. Data represent mean ± SE values of the four experiments. In (**C**), distribution of the fibre lengths measured on all the cells from four experiments are represented. (**D**) Live imaging analysis of the cells transfected with control (upper) or Prom1-expressing (lower) plasmids. Images were shown with 15 minute-intervals, starting at 24 hours after the Prom1 transfection. See also Supplementary Movie S1A and B. (**E**–**H**) The membrane extensions were mainly formed at the rear side against the direction of the migration. (**E**) The definition of the front and rear sides against the cell movement. (**F**) Focused images of the membrane extensions at the front (upper images) and at the rear (lower images) sides of the cell. (**G**,**H**) Quantitative data for the number (**F**) and length (**G**) of the fibres.

In order to characterise the Prom1 protein, we performed an overexpression analysis of Prom1 tagged with YFP in hTERT-RPE1 (RPE1) cells. At 24 hours post-transfection, we observed more than 20 membranous fibres per cell on the cell surface, each with a length of more than 20 μm, on the cell surface, which were not formed in the control YFP-transfected cells (Fig. 1A–C). Moreover, the overexpressed Prom1 protein was localised to these aberrantly formed fibres (Fig. 1A).

We next attempted to characterise the fibres, and performed a live-cell imaging analysis. The Prom1-transfected cells were cultured for 24 hours, and were subjected to sequential snapshots for 2 hours, with a 5 minute-interval (Fig. 1D; supplementary Movie S1A,B). As a result, the cells transfected with *Prom1* randomly moved almost to the same extent as the control GFP-transfected cells did, and longer and a larger number of fibres were found at the rear side than at the front side of the cells to the direction of the movement (Fig. 1E–H). This finding suggests that a multiple types of the fibres were formed by the overexpression of Prom1.

### Formation of the fibres on the membrane by Prom1 is independent from that of actin or tubulin polymerisation, but dependent on cholesterol synthesis

**Figure 2:**
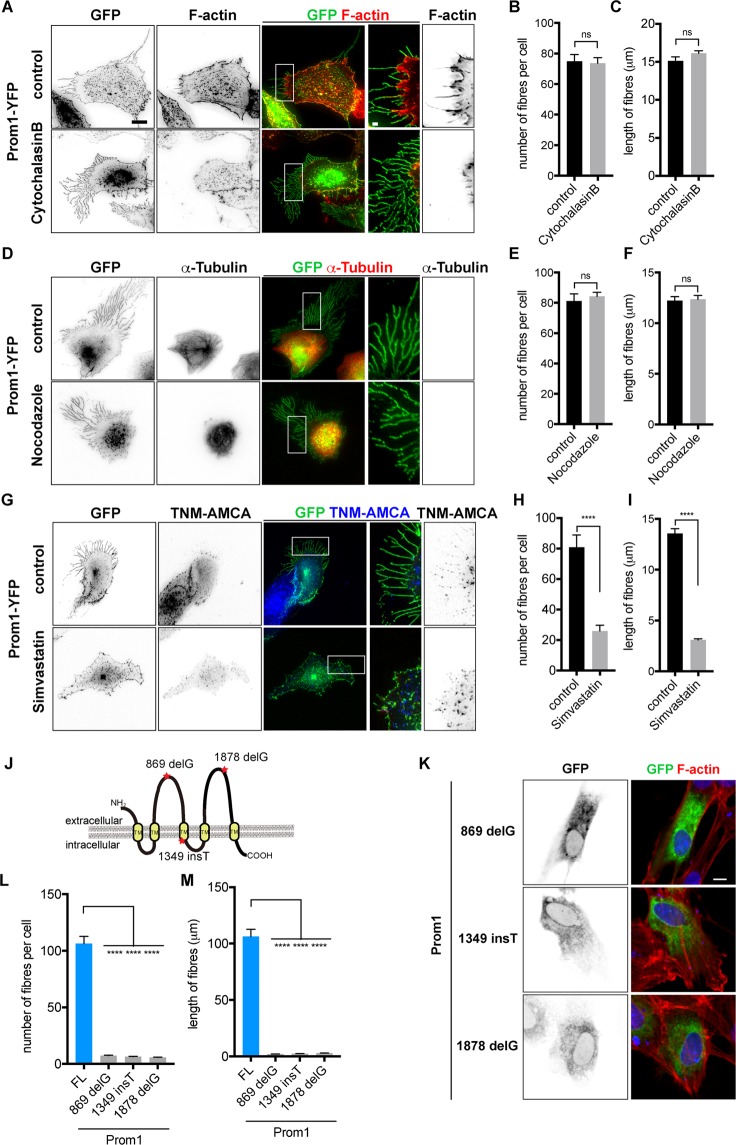
Cell membrane extensions induced by Prom1 are enriched in cholesterol. (**A**–**I**) Formation of the Prom1-induced fibres is independent from Actin (**A**–**C**) or α-Tubulin (**D-F**) polymerisation, but is dependent on cholesterol (**G**–**I**). RPE1 cells were administered with DMSO (control), 10 µM of cytochalasin B (A), 20 µM of nocodazole (**D**) or 1 µM of simvastatin (**G**). The expression plasmid of *Prom1-YFP* was transfected in 6 hours after the application, and cells were incubated for further 24 hours in the presence of the indicated drugs. Cells were analysed by staining with GFP (**A**,**D**,**G**) and phalloidin (**A**), α-tubulin (**D**) antibodies or TNM-AMCA (**G**). Enlarged images corresponding to the white squares are shown in two right panels. (**B**,**C**,**E**,**F**,**H**,**I**) The numbers (**B**,**E**,**H**) and lengths (**C**,**F**,**I**) of the fibres were quantified. The experiments were repeated four times, in each of which 20 cells were analysed. Data represent mean ± SE of these four experiments. (**J**–**L**) The overexpression of Prom1 mutants derived from the RP patients fail to form the extensive structure. (**J**) A schematic representation of Prom1 mutations. The deletion at the 869th guanine nucleotide (869 delG), the insertion at the 1349th thymine (1349 insT) and the deletion at the 1876th guanine nucleotide (1876 delG) lead to the precocious stop codon immediately downstream of the mutation. Nucleotide count is enumerated from the start codon ATG. (**K**) These mutant constructs were transfected into the cells and analysed with GFP antibody and the phalloidin staining. The numbers (L) and lengths (M) of the fibres were quantified. The experiments were repeated four times, in each of which 20 cells were analysed. Data represents the mean values and SE (error bars) of these three experiments. Statistical analyses were performed with unpaired t-tests between the cells with the Prom1FL condition (Fig. 1B) and each construct. Scale bar, 10 µm (**A**,**D**,**G**,**K**), 1 µm (**A**,**D**,**G**; two right panels).

As the extensive structures on cell membrane often contain supporting cytoskeletal components: actin (for cytonemes and retraction fibres) and microtubules (for cilia)^1^, we assessed whether the formation of the membrane extensions is dependent on either of these proteins, and treated the cells with cytochalasin B and nocodazole in order to block actin polymerisation and microtubule formation, respectively. Neither of these treatments perturbed fibre formation upon the transfection of Prom1-YFP, despite actin polymerisation (Fig. 2A–C) and microtubule formation (Fig. 2D–F) being considerably disturbed. These findings revealed that the fibres formed by Prom1 are independent of these major cytoskeletal components with respect to both the structure and the initialisation of formation.

Previous studies have reported that Prom1 is a cholesterol-binding protein^15,20,21^. Therefore, we investigated whether cholesterol is an essential component for membrane extension, and treated the cells with the cholesterol-synthesising inhibitor Simvastatin^22^. The inhibitory effect was confirmed using a fluorescein sterol probe TMN-AMCA^23^, and fibre formation was completely abolished (Fig. 2G–I). This suggests that the cholesterol accumulation is required for fibre formation induced by Prom1.

Various mutations have been found in the Prom1 gene of the RP patients, resulting the production of truncated Prom1 polypeptides (Fig. 2J)^8,10,13^. We therefore asked if these mutant forms of Prom1 correlate with fibre formation, and overexpressed them in the cells. We found that neither of them formed the membrane extensions (Fig. 2K–M), suggesting that fibre formation and photoreceptor deformation are associated with each other.

Thus the Prom1 activity on cell morphology is exerted via direct rearrangement of the membrane components, and does not absolutely require modulation of microtubules or actin.

### The five amino acids located at the carboxyl terminus are responsible for fibre formation

**Figure 3:**
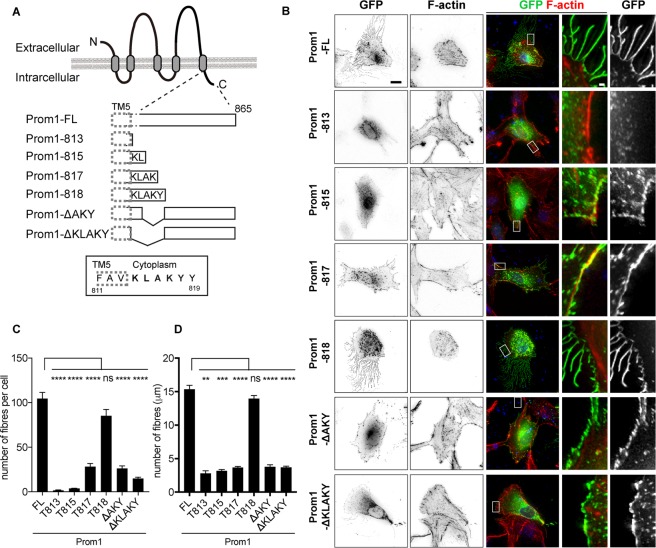
The five amino acids in the carboxyl terminal region are essential for the formation of the cell membrane extensions. (**A**) A schematic representation of the Prom1 protein and its deletion mutants. Prom1 protein used in this study comprises 865 amino acid residues, and the amino terminus (N) and carboxyl terminus (**C**) are located at the extracellular and intracellular regions, respectively. The three amino acids surrounded by a dotted line are in the fifth transmembrane domain (TM5). (**B**) Representative images of the cells transfected with each deletion mutant. The expression plasmids conveying *Prom1-FL* (Full-length of Prom1), *Prom1-813* (as indicated in (**A**)), *Prom1-815*, *Prom1-817*, *Prom1-818*, *Prom1-ΔAKY* or *Prom1-ΔKLAKY* were transfected into the cells, and the cells were analysed by staining with GFP antibody and with phalloidin at 24 hpt. On each row, the right two panels represent magnified images of the areas surrounded by dotted rectangles in the centre images. Scale bar, 10 µm (two left panels), 1 µm (two right panels). (**C**,**D**) Quantitative data for (**B**). The numbers (**C**) and lengths (**D**) of the fibres were counted and measured, respectively. The experiments were repeated four times, in each of which more than 20 cells were analysed. Data represent mean ± SE of these four experiments Statistical analyses were performed with unpaired t-tests between the cells with the Prom1FL condition and each construct.

Next we asked which amino acids are responsible for fibre formation on membrane. Since most Prom1 mutations in the RP patients result in the production of a polypeptide lacking its carboxyl-terminal region (Fig. 2J), we constructed a series of Prom1 truncation mutants speculating that the responsible amino acids would reside in the carboxyl terminus (Fig. 3A).

Overexpression of the Prom1 deletion mutant whose translation ends at the 813th amino acid (Fig. 3A–D) did not form any fibres, presumably because it did not go to the plasma membrane. Nevertheless, when the deletion mutant that contains the five amino acids KLAKY (Lysine-Leucine-Alanine-Lysine-Tyrosine; Prom1-818) was transfected into the cells, the number and the length of the fibres were essentially the same as those formed upon the full-length Prom1 transfection (Fig. 3A–D), whereas the constructs that comprised a part of the KLAKY motif (Prom1-815 and 817; Fig. 3A) led to the formation of incomplete fibres. Conversely, the construct containing the full-coding regions except for the AKY amino acid stretch form generated fewer fibres, and the construct without KLAKY failed to induce fibres entirely (Fig. 3A–D). In these experiments, all the mutants except for Prom1-813 were localised to the plasma membrane (Fig. 3B). Together these findings suggest that these five amino acids are responsible for fibre formation. We further evaluated whether the last tyrosine (Y818) requires phosphorylation for the complete activity of fibre formation, and transfected a construct in which the tyrosine was replaced with phenylalanine (Y818F). However, fibre formation was comparable with that with Prom1-FL (supplementary Fig. 1A). Thus, phosphorylation at this site is unlikely to be necessary for fibre formation.

Taken together, the five amino acids located immediately downstream of the fifth transmembrane domain are essential for fibre formation on the membrane.

### Rho/ROCK signalling is required for the fibre formation by Prom1

We next explored the essential factors that mediate the fibre formation by Prom1. As PI3K^17^ and the tyrosine kinases Src and Fyn^16^ are essential for self-renewal and proliferation of glioma cells, we observed fibres formed upon the Prom1 transfection in cells pre-treated with LY294002^24^ or CGP77675^25^, pan-PI3K and Src inhibitors, respectively. However, no effect of these chemical treatments on fibre formation was observed (supplementary Fig. 1B,C). Moreover, the substitution mutant Y828F, which abolishes essential phosphorylation for the Src signalling activation^17^, was as active as Prom1-FL regarding the fibre formation (supplementary Fig. 1D). This observation suggests that Prom1 has distinct downstream branches, and the membrane extensions formed by Prom1 are induced via differing signalling mediator(s) from those previously reported.

In order to identify novel downstream effectors of Prom1, we evaluated fibre formation following treatment with a number of other cell-signal inhibitors. We focussed inhibitors of the small GTPases, including Rho, Rac and Cdc42, as these GTPases are often involved in fibre formation^26^.

**Figure 4:**
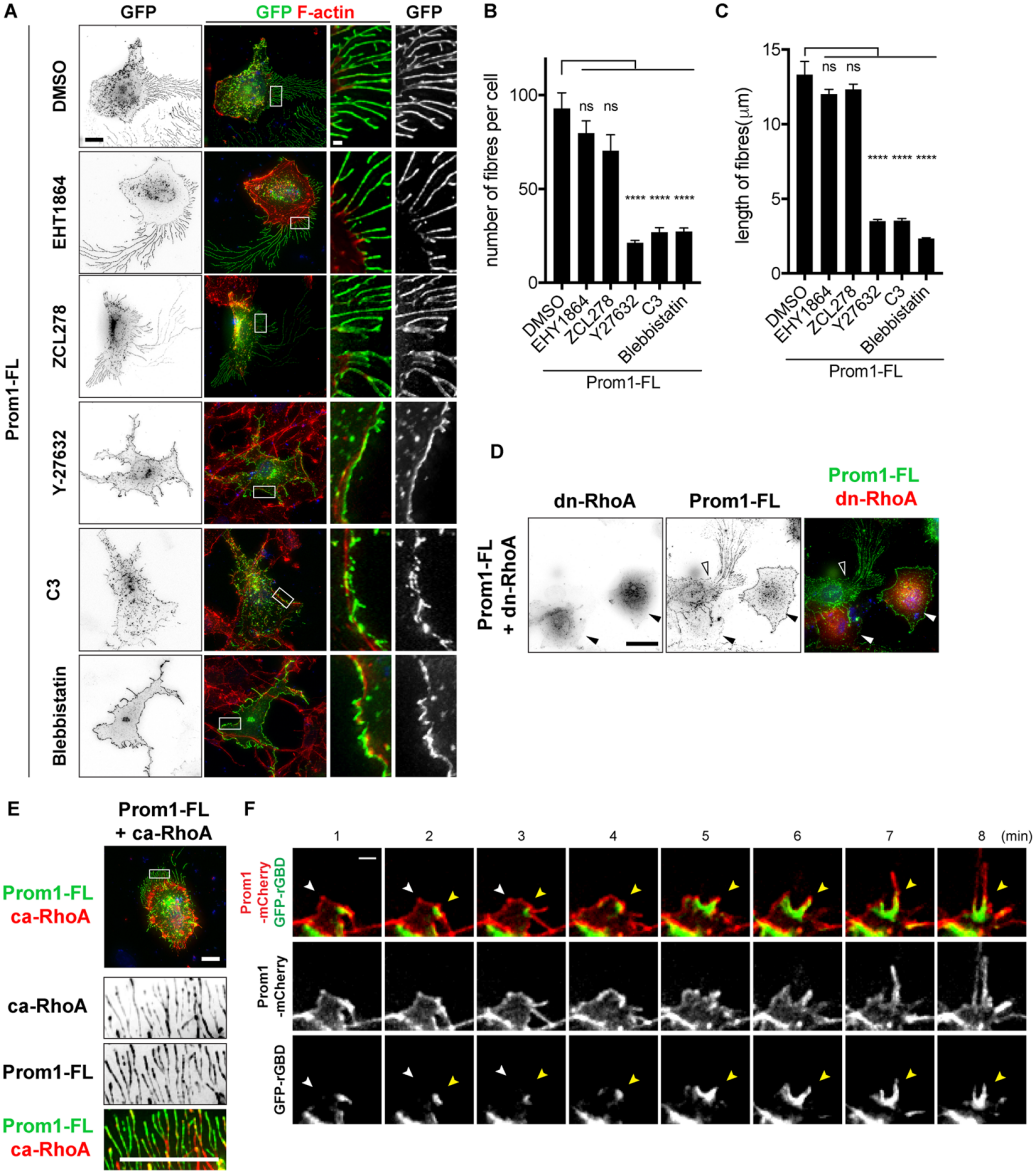
Rho/ROCK signal is essential for fibre formation induced by Prom1. (**A**) The inhibitors targeting the small GTPases (100 nM of EHT1864 for Rac, 50 µM of ZCL278 for Cdc42, 20 µM of Y-27632 for Rho and Rac, 0.5 µg/ml of C3 for Rho, 10 μM of Blebbistatin for Myosin II) were treated for 6 hours and the expression plasmid conveying *Prom1-YFP* was transfected. Cells were analysed with GFP antibody or phalloidin at 24 hpt. (**B**,**C**) Quantitative data for (**A**). The numbers (**B**) and lengths (**C**) of the fibres were counted and measured, respectively. The experiments were repeated four times, in each of which 20 cells were analysed. Data represent mean ± SE of these four experiments. (**D**) The activation of RhoA is essential for fibre formation by Prom1. The plasmid conveying *myc-tagged dominant-negative version of RhoA* (*dn-RhoA*) was co-transfected with *Prom1-FL*. Staining was performed with GFP (for Prom1), myc (for dn-RhoA) antibodies and phalloidin. Outlined arrowheads represent the cell that got the transfection of Prom1, but not dn-Rho. (**E**) The active Rho coincides with Prom1 in the membrane extensions. The plasmid conveying *myc-tagged constitutively-active version of RhoA* (*ca-RhoA*) was co-transfected with *Prom1-FL*. Staining was performed with GFP (for Prom1), myc (for dn-RhoA) antibodies and phalloidin. Enlarged images corresponding to the white squares are shown in the bottom three panels. (**F**) The fibres are formed at the point where Prom1 and active-Rho encounter with each other. The plasmids conveying *GFP-rGBD* and *Prom1-mCherry* (**F**) or control-*mCherry* (**G**) were co-transfected and time-lapse imaging was performed for 6 minutes at 24 hpt, focusing on the initial points of the fibre formation. Yellow and white arrowheads indicate the points with high (where Rho is active) and low GFP (where Rho is inactive) intensities, respectively. 16 fields were analysed, and representative images are shown. See also Supplementary Movie S2. Scale bars, 10 µm (A,E), 1 µm (two right panels), 20 µm (**D**), 1 µm (**F**,**G**).

While EHT1864^27^ and ZCL278^28^, selective inhibitors for Rac1 and Cdc42, respectively, had no effect on fibre formation by Prom1 (Fig. 4A–C), we observed that the ROCK inhibitor Y-27632^29^ substantially reduced the number and the length of the fibres (Fig. 4A–C). As the ROCK inhibitor affects both Rho and Rac signals, we used C3, a membrane-permeable recombinant protein that specifically blocks the Rho signal^30^ to disambiguate between the two molecules. The result showed that C3 had a similar effect to that of Y-27632 (Fig. 4A–C), suggesting that Rho is essential for the fibre formation by Prom1.

We further examined the potential functions of ROCK1/2 in the Prom1-induced fibres, and knocked-down their expression by *si-RNAs* (Supplementary Fig. S2). This knockdown resulted in the perturbation of the fibre formation by Prom1 (Supplementary Fig. S2A–C), confirming that ROCK1/2 are essential for the fibre formation. Moreover, the reduction of phosphorylation of MLC2 was observed by western blotting, and overexpression of Prom1 did not compensate for this reduction (Supplementary Fig. S2D). Thus, the results suggest that ROCK1/2 are essential mediators for the Prom1-induced fibre formation. Furthermore, treatment with Blebbistatin^31^, an inhibitor targeting Myosin II, of the cells also strongly blocked the fibre formation (Fig. 4A-C), supporting the idea that Rho/ROCK-mediated signalling pathway is essential for the fibre formation by Prom1.

We next investigated the relationship between Rho and Prom1. Consistent with the Y-27632 and C3 treatments (Fig. 4A), the co-transfection of the dominant-negative RhoA in combination with Prom1 blocked fibre formation (Fig. 4D), suggesting that RhoA is essential for the fibre formation induced by Prom1. Conversely, the constitutively-active RhoA (ca-RhoA) is localised to the fibres formed by Prom1 (Fig. 4E).

We next investigated where Prom1 initiates the membrane extensions, and in this analysis, we speculated that the sites where these two factors encounter each other would be the initial locations of the fibre formation. To test this hypothesis, we highlighted the initial moment when the fibre was formed. We co-transfected *GFP-rGBD*^32^, which visualises active-Rho, together with *Prom1-mCherry* into the RPE-1 cells, and examined the individual proteins via time-lapse analysis. Prom1-mCherry was localised at the membrane and active-Rho was found randomly in the cells including at the plasma membrane. Importantly, the fibre formation was initiated at the membranous point where active Rho and Prom1 encountered (25/31 cases; Fig. 4F, yellow arrowheads; supplementary Movie S2). On the other hand, at the areas where only Prom1-mCherry was found (and no active-Rho was found), no fibre formation initiated (0/36 cases; Fig. 4F, white arrowheads; Supplementary Movie S2). Thus, these findings suggest that the formation of the membrane extensions by Prom1 is mediated by the small GTPase RhoA, and initiates at the sites where Prom1 and active-Rho encounter.

Although Prom1 and active-Rho are colocalised with each other (Fig. 4F), we were unable to get the proteins to co-immunoprecitate one another suggesting that Prom1 does not physically bind to or activate Rho (supplementary Fig. S2). This suggests that Rho is activated by another triggering factor(s), potentially RhoGEFs (Rho family specific GDP-GTP guanine exchanging factors), and interacts with Prom1 weakly or transiently.

### Prom1 drives chloride ion efflux upon intracellular calcium ion uptake

The high-dimensional structure-based homology search algorithm HHPred^33^ predicted that Prom1 is structurally similar to the membrane proteins TTYH1/2 (supplementary Fig. S4A)^34^. Overexpression of TTYH2 in the RPE cells induced membrane extensions in a manner similar to Prom1 (supplementary Fig. S4B), suggesting that Prom1 and TTYH2 have functional similarities. As the TTYH-type receptors are known to act on calcium-activated chloride currents^35^, we hypothesised that Prom1 has a similar function.

To address this question, we extracted mouse embryonic fibroblast (MEF) cells from wild-type or *Prom1* gene-deficient (*Prom1* knockout; *Prom1*KO) embryos^14,36^ and overexpressed the expression plasmids of Prom1-FL or Prom1-ΔKLAKY into the MEF cells. As the result, we observed the fibre formation by Prom1-FL, with a significantly reduced number and shorter fibres by Prom1-ΔKLAKY (supplementary Fig. S5). This observation suggested the MEF cells have similar intracellular systems as the RPE-1 cells do.

**Figure 5:**
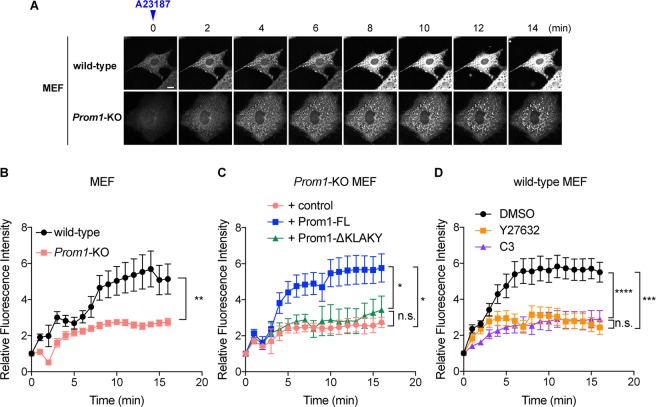
Prom1 modulates the chloride conductance upon intracellular calcium uptake. (**A**,**B**) The calcium-activated chloride efflux is perturbed in the *Prom1*KO MEFs. (**A**) The temporal change in fluorescein intensities of MQAE was measured. The wild-type and *Prom1*KO MEF cells were incubated with low-chloride Kreb’s medium (see materials and methods) and the intracellular calcium uptake was provoked by adding 5 µM of the calcium ionophore A23187 onto the medium (time 0). The temporal change of the fluorescein intensity was imaged at 1 min intervals up to 15 min after the ionophore treatment under the confocal microscope. Representative images are presented. Scale bar, 10 μm. (**B**) Quantitative data for (**A**). Eight cells were selected from each of wild-type and *Prom1*KO cells, and the fluorescein intensities at each time point were quantified. Data are represented as the mean values ± s.e.m. (**C**) The chloride efflux is rescued by overexpression of Prom1-FL, but not by Prom1-ΔKLAKY in the *Prom1*KO MEF cells. The expression plasmids conveying *Prom1-FL* or *Prom1-ΔKLAKY* were transfected into the *Prom1*KO MEF cells, and cells were incubated in the presence of MQAE. The transfected cells were identified by YFP expression, and the fluorescein intensities from each transfection were traced. 3 cells for control, 7 cells for Prom1-FL and 8 cells for Prom1-ΔKLAKY were measured. (**D**) The chloride efflux is perturbed upon the treatment with Rho inhibitors Y27632 and C3. Wild-type MEF cells were treated with DMSO (control), 20 μM of Y-27632 or with 0.5 μg/ml of C3 for 2 hours at the same time of the MQAE treatment and were subjected to the fluorescein measurement as in (**B**,**C**). 9 cells for DMSO, 12 cells for Y27632 and 10 cells for C3 were analysed.

By using this system, we measured the temporal changes in the intracellular chloride ion level upon calcium uptake by using the chloride-sensitive fluorescent indicator MQAE (N-(ethoxycarbonylmethyl)-6-methoxyquinolinium bromide). As MQAE is quenched by chloride ions, the fluorescein intensity is reciprocal to the intracellular chloride ion concentration. Once intracellular calcium uptake was provoked by the calcium ionophore A23187, significant chloride efflux was observed in the wild-type cells within several minutes (8 min; Fig. 5A,B, Supplementary Movie S3A). In contrast, the extent of the efflux was reduced by approximately 50% in the *Prom1*KO cells (8 min; Fig. 5A,B, Supplementary Movie S3B), thus chloride ions remained at a high level in the cells. A similar result was obtained in another analysis in which the cell mass was measured (supplementary Fig. S6A). Importantly the extent of calcium uptake upon the A23187 treatment was comparable (supplementary Fig. S6B), suggesting that the perturbation of chloride ion efflux was not a secondary effect resulting from a change in calcium influx. Collectively, these observations suggest that Prom1 modulates dynamic intracellular chloride current upon calcium uptake.

Furthermore, we investigated whether this efflux perturbation in the *Prom1*KO cells was rescued upon transfection of the wild-type Prom1 and Prom1-ΔKLAKY. When we transfected Prom1-FL in the *Prom1*KO cells, the chloride efflux was found to be restored to the same level as in the wild-type MEF cells (Fig. 5C, supplementary Fig. S7A and Supplementary Movie S4A–D). However, this outflow failed to occur upon the transfection of Prom1-ΔKLAKY, suggesting that the amino acids stretch KLAKY (Fig. 5C, Supplementary Fig. S7A and Supplementary Movie S4E,F) was essential for regulation of chloride efflux. Moreover, wild-type MEF cells pre-treated with Rho/ROCK inhibitors Y-27632 or C3 perturbed the chloride efflux upon the calcium uptake (Fig. 5D, supplementary Fig. S7B and Supplementary Movie S5A–C). Collectively, these findings suggest that the function of Prom1 as the membrane morphology modulator and the chloride ion current regulator are closely associated with each other.

## Discussion

In this study, we have demonstrated that Prom1 triggers the formation of cholesterol-dependent membrane extensions (Figs. 1,2) through its carboxyl-terminal region (Fig. 3). Moreover, the activation of the small GTPase RhoA mediates the fibre formation (Fig. 4). We have also showed that Prom1 is structurally similar to TTYHs, proteins involved in the calcium-activated chloride currents^35,37^, and is involved in the chloride current activated by calcium uptake (Fig. 5). Together, these findings suggest that Prom1 plays essential roles for cell morphology and regulation of intracellular ion balance.

The fibres were formed not only in RPE-1 cells, but also in ARPE-19 (Supplementary Fig. 1E) and MEF cells (supplementary Fig. 5), which are distinct types of cells, suggesting that the role of Prom1 function in fibre formation is not limited to RPE-1 cells. While the fibres contain F-actin, the formation of membrane extensions by Prom1 is not dependent on F-actin or α-tubulin polymerisation, but on membrane cholesterol rearrangement (Fig. 2). Previous^9,20^ and recent^38^ reports have demonstrated that the structures induced by the overexpression of Prom1 have characteristics of the microvilli. Moreover, it has been shown that Prom1 resides also in other protrusive structures^15,21,39^ including cytonemes^38^ and cilia^40^, where cholesterol is an important ingredient^41,42^, and this fact is consistent with our current findings that cholesterol is essential for the fibre formation in RPE-like cells. However, our live-imaging analysis of the Prom1-transfected cells suggested that more types of membrane extensions can be formed by the overexpression of Prom1. Particularly, some of the extensions seem to be retraction fibres; actin-enriched projections formed at the rear edge of the moving cells^2,43^, rather than lamellipodia or filopodia, formed at the leading edge^43^. This assumption is reasonable because the extensions found in this context is dependent on Rho/ROCK rather than Rac and Cdc42 (Fig. 4A)^43^. Moreover, it has been shown that the osmotic pressure could cause cell retractions^44^, which is consistent with our finding that Prom1 is involved in the dynamics of intracellular chloride ions (Fig. 5)

In contrast to these observations, *in situ* RPE cells are not believed to be motile, so it is difficult to evaluate the physiological significance of the retraction fibres, at least in the retinal context. However, the apical surface of RPE are covered with microvillus-like apical processes and the basal surface is elaborated into a complex actin-rich labyrinth composed of basal infoldings^45^. It has been shown that the structure of the latter is dependent upon osmotic changes^45^. Therefore, the extentions we observed in culture cells may provide clues to what is occurring *in vivo*. Detailed characterization (e.g., an ultrastructural analysis) of the structure where Prom1 is localized is warranted to reveal the Prom1 functions in the physiological context.

The five amino acid residues in the carboxyl-terminal region, conserved between human and mouse, are essential for the fibre formation (Fig. 3). These amino acid residues are lacking in most RP patients harbouring mutations in the *Prom1* gene^8,10,13^, suggesting that the formation of membrane extension is, at least, one of the essential functions of Prom1 for retinal homeostasis.

We have further elucidated that the membrane domain where the active-Rho and Prom1 colocalise is the site where the extension initiates. Since active-Rho recruits cholesterol to form lipid rafts^46^, Prom1 and active-Rho act in conjugation with each other, and form the fibres where Prom1 can exert its functions, including autophagy^6,47,48^, migration^49–52^ and metastasis^53^.

Whilst active-Rho and Prom1 colocalise, Rho is not activated by Prom1 (supplementary Fig. S3A,B), and we could not identify a direct interaction by a coprecipitation assay (supplementary Fig. S3C). Therefore, the regulation of Rho activation is independent of the abundance or localisation of Prom1, and the colocalisation of Prom1 and active-Rho is mediated by another molecule. Further analyses, including a genome-wide screen searching for the interacting proteins of prom1 or Rho, will elucidate other essential proteins necessary for the fibre formation. Reconstitution of membrane curvature with active-Rho, Prom1 and lipid is also a possible trial.

TTYH1/2 have high structural homology to Prom1, and also induce fibre formation upon overexpression (supplementary Fig. S4A,B). Moreover, TTYH1/2 have been shown to be involved in the calcium-activated chloride current in Chinese Hamster Ovary (CHO) cells^35,37^. The physiological significance of TTYHs in retinal homeostasis remains unclear, as no knockout mice of *TTYH1/2* have been analysed to date. Nevertheless, *TTYH* genes have been shown to be expressed in the retina^54–56^. Moreover, dynamic ionic current is apparently crucial for retinal functions^57^. With respect to chloride current triggered by intracellular calcium uptake, the critical roles of calcium-activated chloride channels (CaCCs) have been demonstrated. Bestrophin^58^ and ANO1 (also called as TMEM16A)^59,60^ are representative CaCCs and have been shown to be essential for the photoreceptor homeostasis; the mutations or deficiencies of those genes recapitulate severe genetic retinopathies including macular degenerations and RPs. However, it seems that Prom1 is not completely redundant with those CaCCs. First, in the retina, Bestrophin and ANO1 are expressed in RPE^61^ and at the tip of photoreceptor axons^62^, respectively, while Prom1 is localised to photoreceptor and RPE cells. Moreover, in our overexpression experiment, no fibres have been found upon overexpression of these genes (supplementary Fig. S4C). Thus the molecular mechanisms by which the chloride current is regulated are partially distinct between Prom1 and these CaCCs.

One major protein that associates with intracellular calcium ion dynamics is rhodopsin. Rhodopsin is a GPCR (G-protein coupled receptor) converting light stimuli to the cGMP activation followed by the intracellular calcium uptake^63^. Rhodopsin is localised to the membrane extensions (Supplementary Fig. S8A) and physically interacts with Prom1 (supplementary Fig. S8B), suggesting that these two proteins act in conjugation with each other. As rhodopsin is activated by light stimuli, it can provoke Ca2^+^ uptake, and Prom1-mediated Cl^−^ current will follow. Future analysis can focus on an *in vivo* calcium ion change, because it will elucidate the importance of the chloride ion efflux regulated by Prom1 at the physiological level.

How the two functions of Prom1 revealed in this study - fibre formation and chloride current modulation - are relevant to each other is so far remains to be elucidated. However, it is clear that these functions are relevant to each other, as a Prom1 mutant (ΔKLAKY) that cannot form membrane extensions (Fig. 3), does not have the activity of chloride ion efflux, either (Fig. 5C). Together the data obtained in this study with previous studies, it is reasonable to speculate that Prom1 assembles the functional membrane molecules, including rhodopsin and cholesterol, with a Rho-dependent manner, and starts the evagination of the cell membrane where Prom1 functions as the chloride ion efflux regulator (supplementary Fig. S8C). A future analysis searching for the interacting proteins of Prom1 will identify proteins that function together with Prom1.

In order to treat genetic retinopathies and identify novel therapies, it is logical to identify both the genetic lesions responsible for the retinal degeneration and their downstream targets. Our present study has identified possible downstream targets in the Prom1 degeneration pathway: Rho, cholesterol and/or chloride ions. Altogether, by applying the current study to the physiological levels, we envisage to provide new insights in developing novel therapeutic methods for intractable hereditary retinopathies.

## Materials and Methods

### Ethical statement on animal experiments

All animal experiments were subjected to the approval of the animal welfare and ethical review panel of Nara Institute of Science and Technology (approval numbers: 1533 and 1810 for animal research, and 311 for genetic modification) and Institutional Animal Care and Use Committee of RIKEN Kobe branch, and all experiments were performed in accordance with relevant guidelines and regulations. *Prom1*KO mice established previously^36^ (CDB0623K: http://www2.clst.riken.jp/arg/methods.html) were reared as a hybrid genetic background of C57BL/6 and CBA/NSlc^14^.

### Cell culture, transfection and Rho activation assay

The human immortalised retinal pigmented epithelium-derived cell lines hTERT-RPE1 (ATCC CRL-4000) and ARPE-19 (CRL-2302) was cultured in high-glucose Dulbecco’s Modified Eagle Medium (DMEM; Wako, Japan) containing 10% FBS (Gibco) supplemented with non-essential amino acids, glutamine and penicillin/streptomycin (Wako, Japan).

While multiple isoforms have been reported for the Prom1 transcripts^7,64^, we employed the isoform encoding 865 amino acids (NCBI accession number NP_006008.1) expressed in the retina and testis, throughout in this study. The amino acid numbers denoted in Fig. 2A are based on this information. The Prom1 constructs were carboxyl-terminally fused with YFP or mCherry as indicated. The coding regions of *TTYH2*^37^, *Best1*^58^ and *ANO1*^59,60^ were isolated by reverse-transcription polymerase chain reaction (RT-PCR), and constructed. DN-Rho and ca-Rho were constructed as described previously^65^.

The plasmids were transfected with Lipofectamine-2000 (Invitrogen). Rho activation assay was performed by using the Rho activation assay kit (Millipore). Immunoprecipition was performed with the magnetic beads conjugated with myc antibody. For RNAi experiments, Silencer select *si-RNAs* targeting *ROCK1* (*si-ROCK1*; s12097), *ROCK2* (*si-ROCK2*; s18162)^66^ and *si-control* (4390843) were purchased from Life Technologies, and 10 nM of *si-RNAs* were transfected into the cells by using Lipofectamin RNAiMAX (Invitrogen).

Antibodies used in this study were; GFP (rabbit; MBL; #598), myc (mouse; CST; #2276 S), HA (mouse; SIGMA; #H9658), phospho-MLC2 (pMLC2; rabbit; Abcam; #ab2480), MLC2 (rabbit; Abcam; #ab79935), α-tubulin (mouse; SIGMA; #T5168). Chemicals were; cytochalasin B (Wako, Japan; #030-17551), nocodazole (SIGMA; #M1404), Simvastatin (Cayman chemical; #10010344), EHT1864 (Cayman chemical; #17258), ZCL278 (TOCRIS; #4794), Y-27632 (Wako, Japan; #251-00511), C3 (Cytoskeleton,Inc; #CT04). LY294002 (Wako, Japan; #129-04861), Blebbistatin (Wako, Japan; # 021-17041), CGP77675 (Cayman Chemical; #21089).

### Immunofluorescence microscopy and fibre formation analysis

Immunofluorescence microscopy was performed as described previously^67^. Briefly, cells were fixed with 4% PFA for 20 min and washed in PBS. After blocking with PBS/1% BSA for 1 h at room temperature, cells were incubated with primary and subsequently with secondary antibodies. During time-lapse imaging, cells were kept at 34-37 °C by a chamber heater.

Fluorescence microscopic analyses were carried out using DeltaVision Elite Microscopy System (GE Healthcare, UK). Z-axial images were taken at 0.2 µm with a 40X objective lens. Deconvolution of images was performed using DeltaVision SoftWoRx software. Captured images were processed with Adobe Photoshop CS5. The numbers and lengths of fibres formed on the cell membrane were measured with ImageJ software and at least 20 cells were analysed on each experiment.

### Intracellular chloride ion measurement on MEF cells

Mouse embryonic fibroblasts (MEF) were prepared from 14.5 dpc (days post-coitum) mouse embryos as described previously^68^, and maintained in DMEM/F-12 (Wako) with 10% of new-born calf serum (MP Biomedicals) supplemented with the antibiotic primocin (Invivogen). For measuring the intracellular chloride ion level, the chloride-sensitive fluorescent indicator MQAE (Dojindo) was used and was used to treat the MEF cells according to the manufacturer’s instruction. Briefly, MEF cells were cultured in the low-chloride medium (Krebs-HEPES buffer; 20 mM HEPES-NaOH (pH 7.3), 128 mM NaCl, 2.5 mM KCl, 2.7 mM CaCl_2_, 1 mM MgSO_4_, 16 mM glucose) and the final concentration of 5 mM of MQAE was added, along with measurement of the basal chloride level. The calcium ionophore (A23187; Sigma) was then added at 5 µM and the temporal change in the chloride ion was measured using LSM 710 confocal microscope (Zeiss) or with the plate reader Tristar2 (Berthold Technologies) at 1 min intervals.

### Structure prediction, images, and data analysis

The homology search based on the secondary structure was conducted using the prediction algorithm HHPred^33^. Images were observed using LSM 710 confocal microscope (Zeiss) or DeltaVision Elite (GE Healthcare) and processed by the Photoshop software (Adobe). Statistical analysis was performed by two-tail t-test using the Prism software (graphpad.com) and *p*-values (*p < 0.05, **p < 0.01, ***p < 0.001) are indicated in each graph.

## Supplementary information

Supplementary Movie.S1A

Supplementary Movie.S1B

Supplementary Movie.S2

Supplementary Movie.S3A

Supplementary Movie.S3B

Supplementary Movie.S4A

Supplementary Movie.S4B

Supplementary Movie.S4C

Supplementary Movie.S4D

Supplementary Movie.S4E

Supplementary Movie.S4F

Supplementary Movie.S5A

Supplementary Movie.S5B

Supplementary Movie.S5C

Supplementary_Information

## Data Availability

All data are available in the main text/figures and in the Supplementary Information.
