# Supplementary material for: Prominin-1 Modulates Rho/ROCK-Mediated Membrane Morphology and Calcium-Dependent Intracellular Chloride Flux
Source: Sci Rep. 2019 Nov 4;9:15911. doi: 10.1038/s41598-019-52040-9 (PMC6828804; doi:10.1038/s41598-019-52040-9)
Supplement: Supplementary file 15 — Supplementary_Information [file 41598_2019_52040_MOESM15_ESM.pdf]

Supplementary Information

Prominin-1 Modulates Rho/ROCK-Mediated Membrane Morphology and Calcium-Dependent Intracellular Chloride Flux

Akiko Hori[1], Kenji Nishide[2], Yuki Yasukuni[1], Kei Haga[1], Wataru Kakuta[1], Yasuyuki Ishikawa[3], Matthew J Hayes[4], Shin-ichi Ohnuma[4], Hiroshi Kiyonari[5], Kazuhiro Kimura[6], Toru Kondo[2,7] and Noriaki Sasai[1]*

1. Developmental Biomedical Science, Division of Biological Sciences, Nara Institute of Science and Technology, 8916-5, Takayama-cho, Ikoma 630-0192, Japan
2. Laboratory for Cell Lineage Modulation, Center for Developmental Biology, 2-2-3, Minatojima-Minamimachi, Chuo-ku, Kobe 650-0047, Japan
3. Department of Systems Life Engineering, Maebashi Institute of Technology, 460-1, Kamisadori-cho, Maebashi, Gunma, 371-0816, Japan
4. Ocular Biology and Therapeutics Unit (ORBIT), Faculty of Brain Sciences, UCL Institute of Ophthalmology, University College London, 11-43 Bath Street, London EC1V 9EL, United Kingdom
5. Laboratory for Animal Resources and Genetic Engineering, RIKEN Center for Biosystems Dynamics Research, 2-2-3, Minatojima-Minamimachi, Chuo-ku, Kobe 650-0047, Japan
6. Department of Ophthalmology, Yamaguchi University School of Medicine, 1-1-1 Minamikogushi, Ube 755-0046, Japan
7. Division of Stem Cell Biology, Institute for Genetic Medicine, Hokkaido University, Kita-15, Nishi-7, Kita-Ku, Sapporo 060-0815, Japan

*Correspondence to Noriaki Sasai
Tel: +81-743-72-5650
noriakisasai@bs.naist.jp

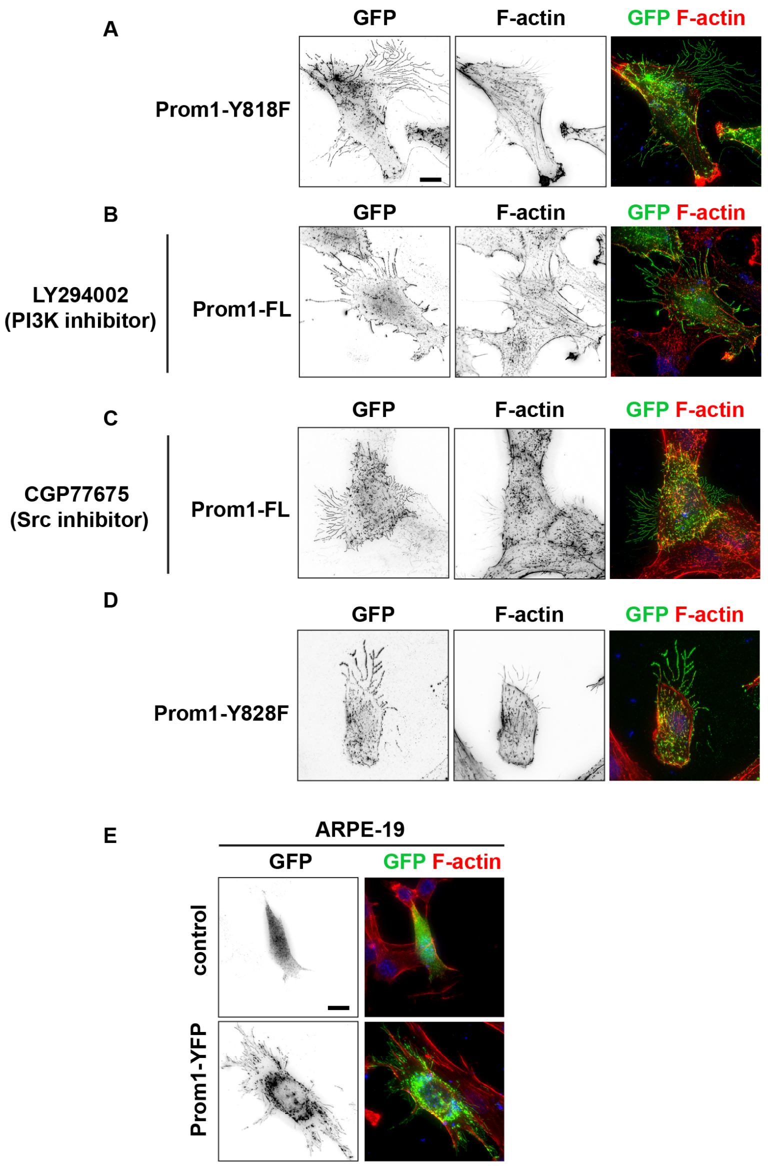

Supplementary Fig. 1 The amino acids related to PI3K or Src in Prom1 are not involved in the cellular morphogenesis. The Prom1 mutants in which the 818th and 828th tyrosines were replaced with phenylalanine (Y818F; A, Y828F; D) were transfected. PI3K and src inhibitors do not have the inhibitory effects on the fibres generated by Prom1. 10 µM of LY294002 (a PI3K inhibitor; B) or and 2 µM of CGP77675 (a Src inhibitor; C) were treated for 6 hours and Prom1-FL was transfected. In all cases, the cells were harvested 24 hours after the transfection and the cell shape was analysed by the GFP antibody and phalloidin staining. (E) Prom1 triggers fibre formation in ARPE-19 cells upon overexpression. The experiment was performed as in Fig. 1A. Scale bar, 10 µm.

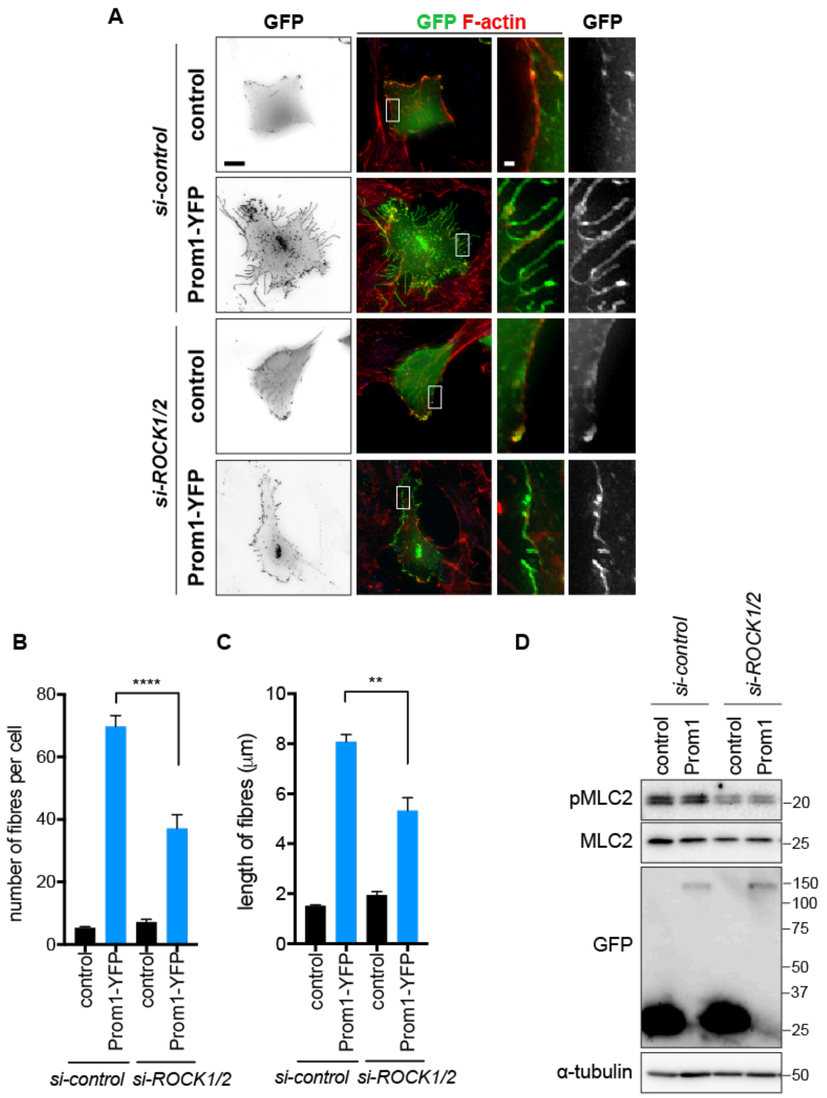

Supplementary Fig. 2 *si-ROCK1/2* block the fibre formation induced by Prom1, and reduce the phosphorylation of MLC2. (A) The control vector or the expression plasmid of *Prom1-YFP* were transfected at 24 hours after the transfection of *si-control* or *si-ROCK1/2*. The cell shape was evaluated at 24 hours after the transfection of the plasmids by staining with GFP antibody and phalloidin. (B,C) Quantitative data for (A). The numbers (B) and lengths (C) of the fibres were counted and measured, respectively. The experiments were repeated four times, in each of which 20 cells were analysed. Data represent mean ± SE of these four experiments. (D) The phosphorylation of MLC2 was reduced by *si-ROCK1/2*. The experiment was performed as in (A), and cells were harvested to be analysed by western blotting with the pMLC2, MLC2, GFP and α-tubulin antibodies.

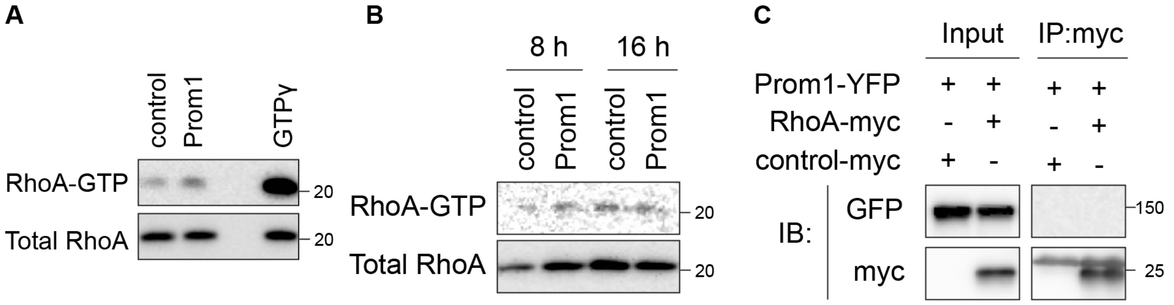

Supplementary Fig. 3 Prom1 does not activate or interact with Rho. (A,B) Rho activation assay. The control vector or the plasmid conveying *Prom1* were transfected into the RPE-1 cells and Rho-activation assay was performed at 24 (A), 8, or 16 hpt (B). (C) Prom1 does not interact with RhoA. Plasmids conveying *Prom1-YFP*, *Rho-myc* and the control vector were transfected as indicated, and immunoprecipitation was performed with the magnetic beads conjugated with myc antibody and detected with the GFP antibody. (B) Quantitative data for (A). The numbers (B) and lengths (C) of the fibres were counted and measured, respectively.

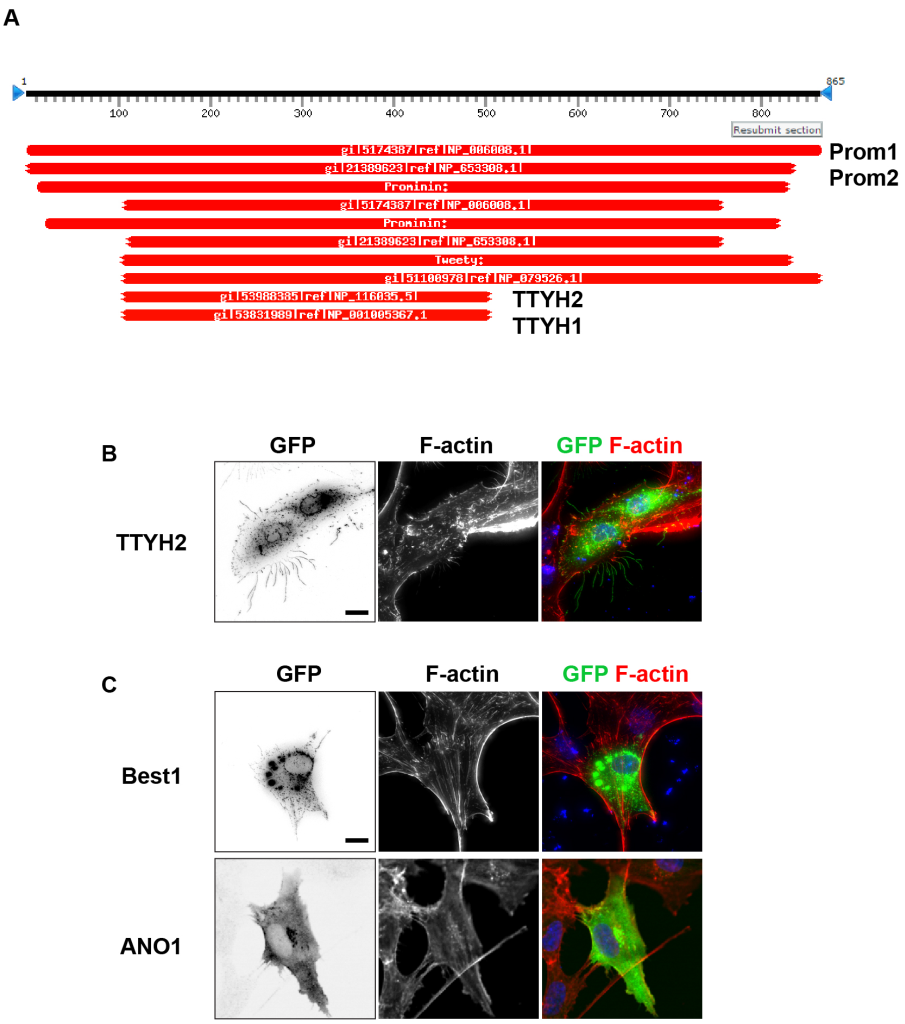

Supplementary Fig. 4 Prom1 is structurally analogous with TTYH proteins, and TTYH has an activity to induce the fibres, as Prom1 does. (A) The outcome of the homology search by using the algorithm HHPred. (B) TTYH2 induces the fibres. The expression plasmids encoding *TTYH2-YFP* were transfected, and the cell morphology was observed 24 hours after the transfection by staining with GFP antibody and phalloidin. Scale bars, 10 µm. (C) Best1 and ANO1, two representative CaCCs, do not induce fibres upon overexpression. Experiments were performed as in (B), except that plasmids encoding *Best1* and *ANO1* were transfected. Scale bars, 10 µm.

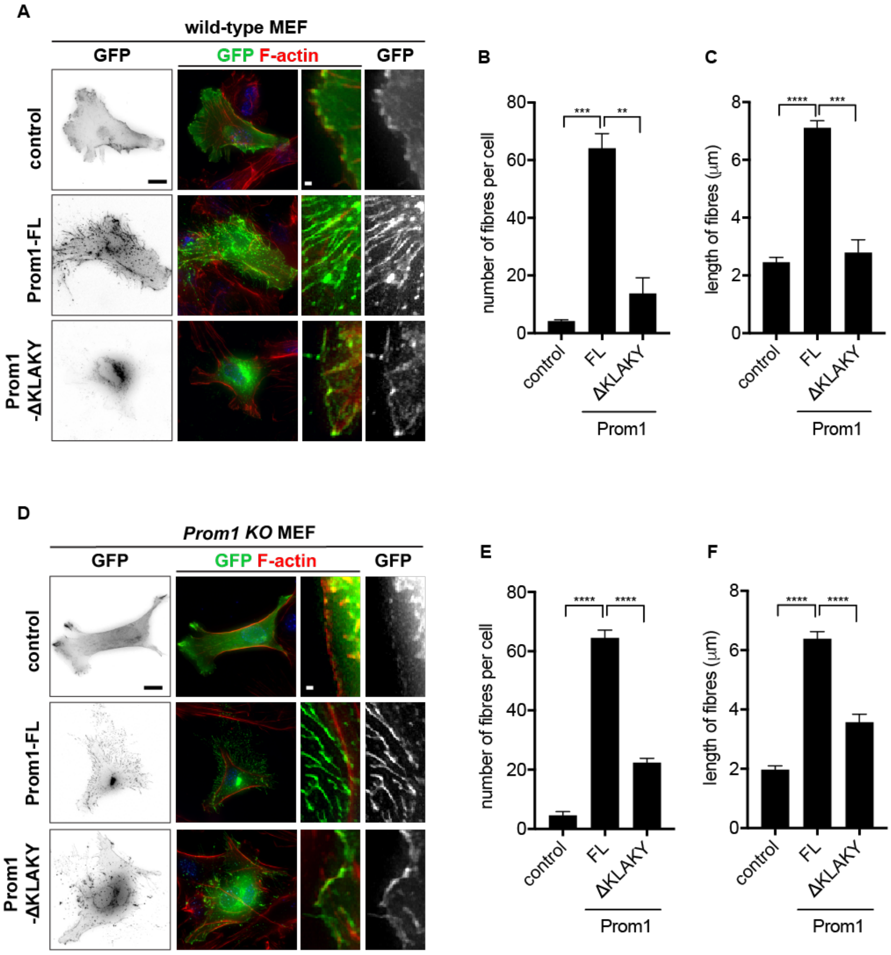

Supplementary Fig. 5 Fibres are formed both in the wild-type and *Prom1*-KO MEF cells by the overexpression of Prom1. (A,D) The control vector or the expression plasmids of *Prom1-FL* or *Prom1ΔKLAKY* were transfected into the wild-type and *Prom1*-KO MEF cells. The cell shape was evaluated at 24 hours after the transfection by staining with GFP antibody and phalloidin. Scale bars, 10 μm (left two panels), and 1 μm (two right panels). (B,C,E,F) Quantitative data for (A) and (D). The numbers (B,E) and lengths (C,F) of the fibres were counted and measured, respectively. The experiments were repeated three times, in each of which 20 cells were analysed. Data represent mean ± SE of these three experiments. Statistical analyses were performed by one-way analysis of variance (ANOVA) followed by Tukey's post-hoc test.

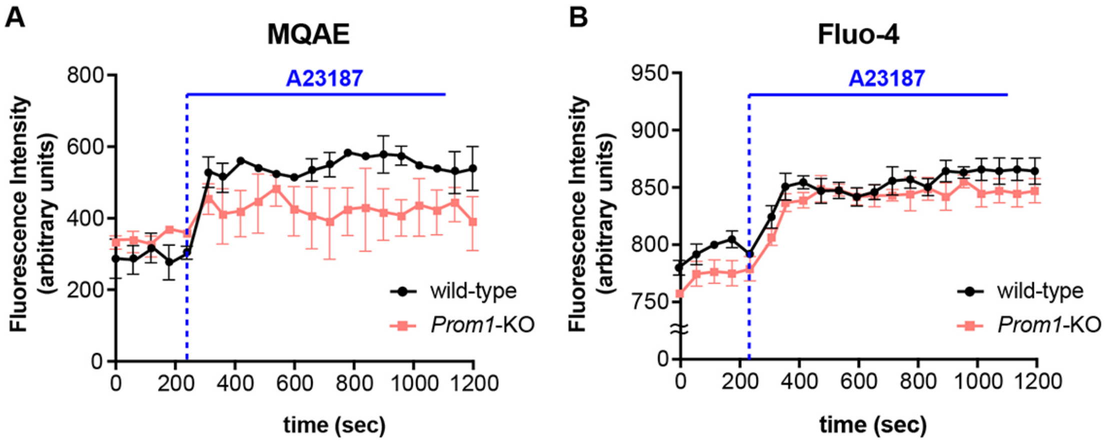

Supplementary Fig. 6 The fluorescein measurement from a plate leader demonstrates similar results as in Fig. 4A, and the intracellular calcium uptake induced by A23187 is comparable in the wild-type and *Prom1*KO cells. (A) The MQAE fluorescein intensities emitted from the cell mass were measured. 1 × 10^3 cells of wild-type or *Prom1*KO MEF cells were plated on a 96-well plate, and were treated with 5 µM of A23187. The measurement was performed at 1 min intervals for 20 min. The measurements were performed thrice for both wild-type and the *Prom1*KO cells and data are represented as the mean values ± s.e.m. (B) Intracellular calcium uptake is not affected in the *Prom1*KO cells upon the treatment with A23187. The experiment was performed as in (A), except that the cells were treated with Fluo-4, a fluorescent calcium indicator.

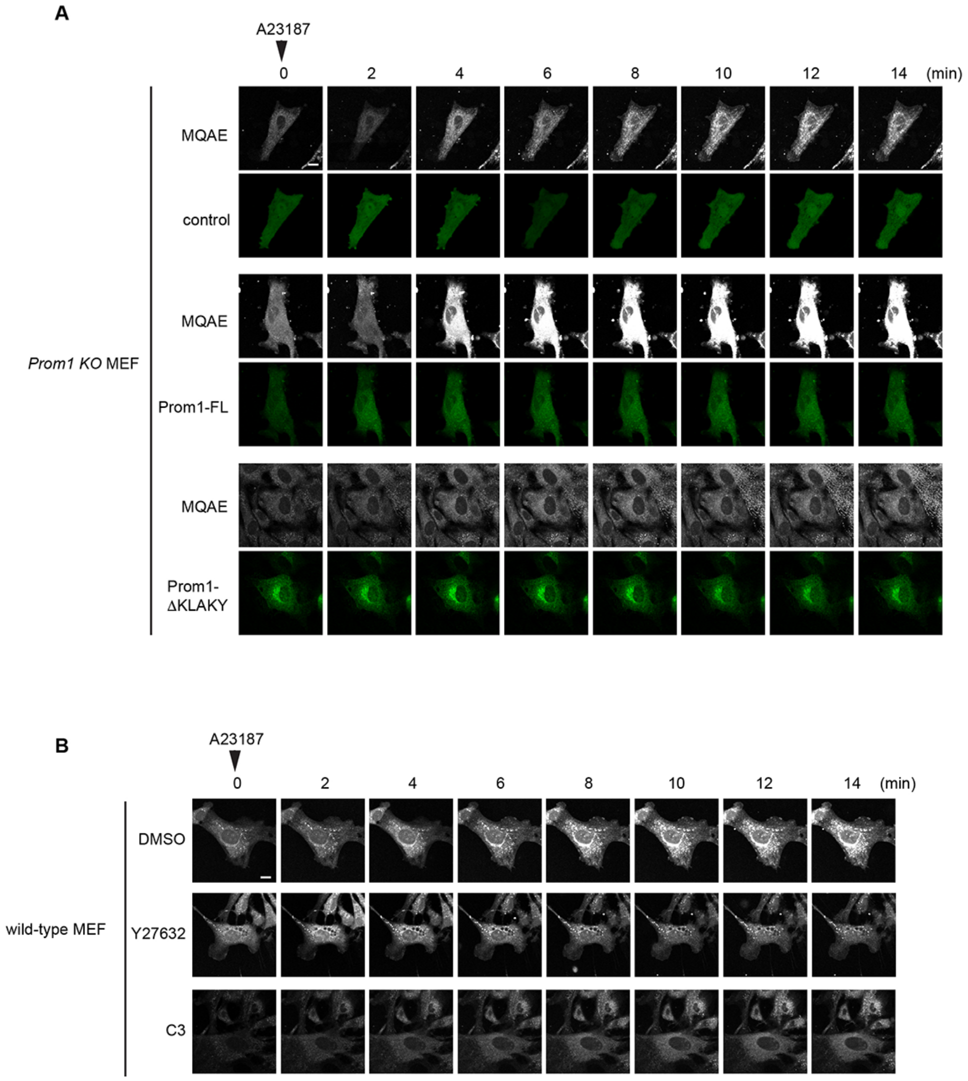

Supplementary Fig. 7 Typical images of temporal fluorescein changes during the culture with MQAE.

The typical images producing Fig. 5C, D are presented. Scale bar, 10 μm.

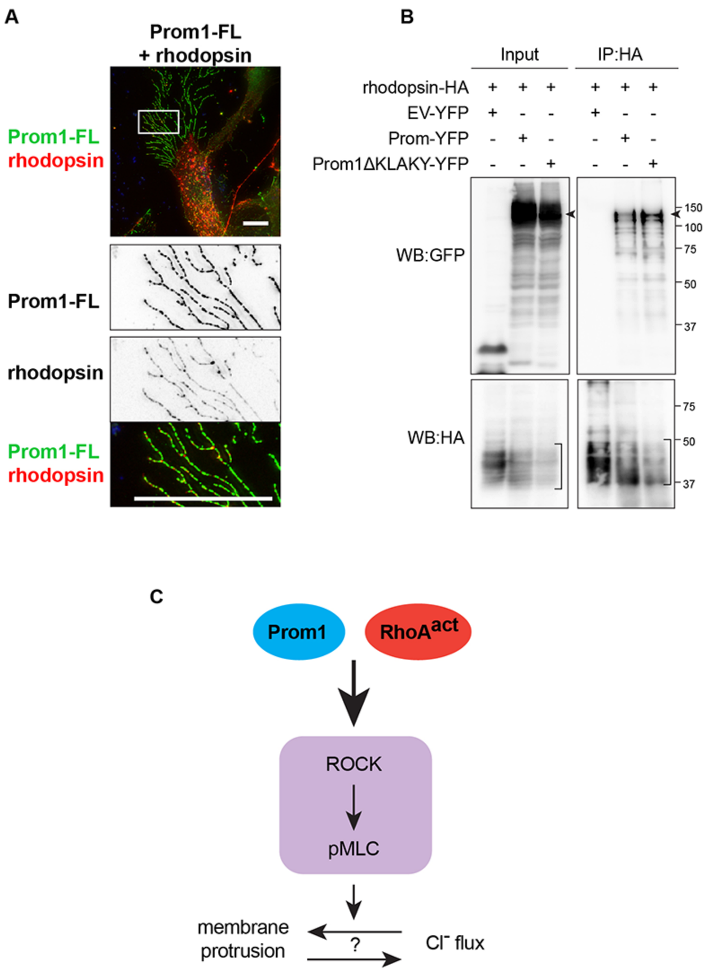

Supplementary Fig. 8 Rhodopsin and Prom1 interact with each other and are co-localised at the fibres. (A) Localisation of rhodopsin in the fibres. The expression plasmids conveying *Rhodopsin-HA* and *Prom1-YFP* were co-transfected into the RPE-1 cells and were harvested to be analysed via immunofluorescence using HA and GFP antibodies. Enlarged images corresponding to the white squares are shown in the bottom three panels. Scale bars, 10 μm. (B) Both Prom1 and Prom1-ΔKLAKY physically interact with rhodopsin. Prom1-FL and rhodopsin-HA were transfected as indicated. Immunoprecipitation and was performed with the magnetic beads conjugated with HA, and the expression was detected with GFP and HA antibodies. (C) Working hypothesis on the Prom1 function. The coincidence of Prom1 and active-Rho activates the intracellular signalling pathway mediated by ROCK1/2 and pMLC, and thereby regulates the fibre formation and the chloride ion flux.

Figure S2

S2D

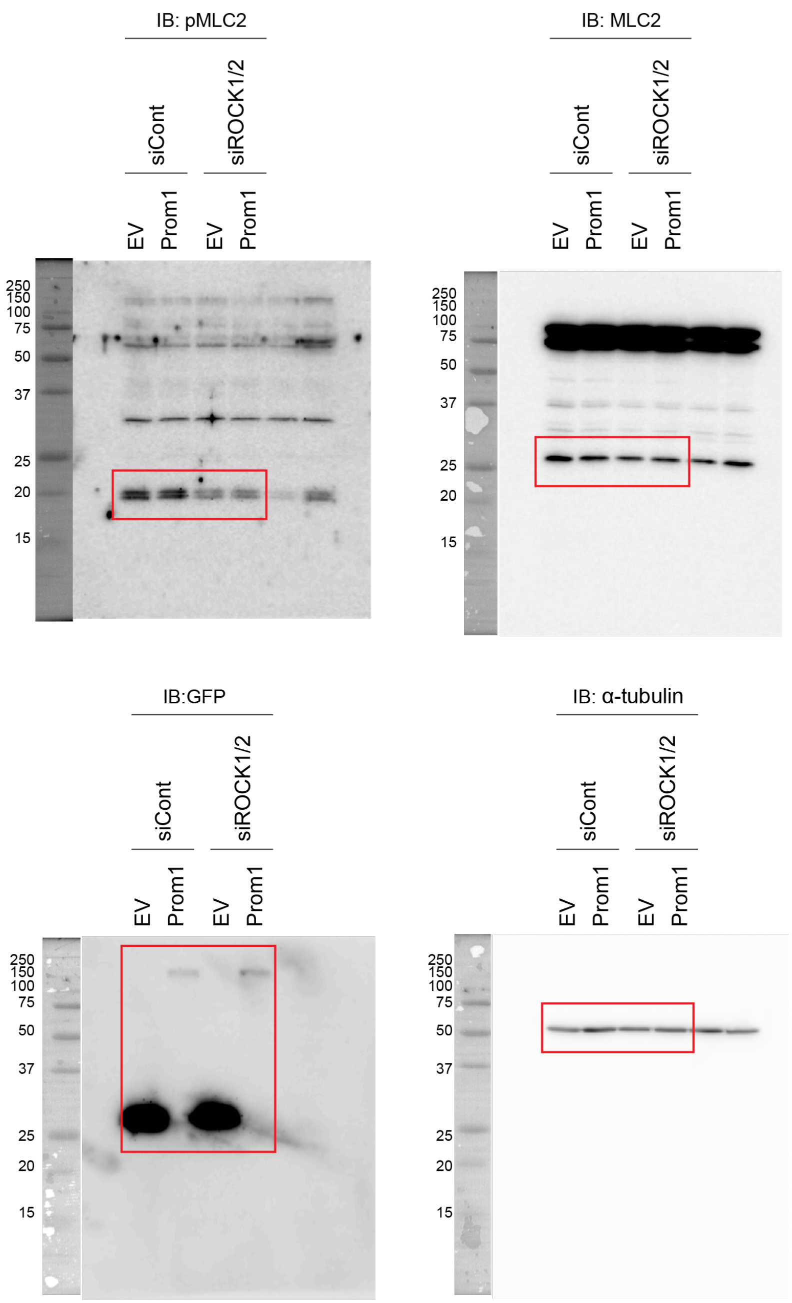

Figure S3

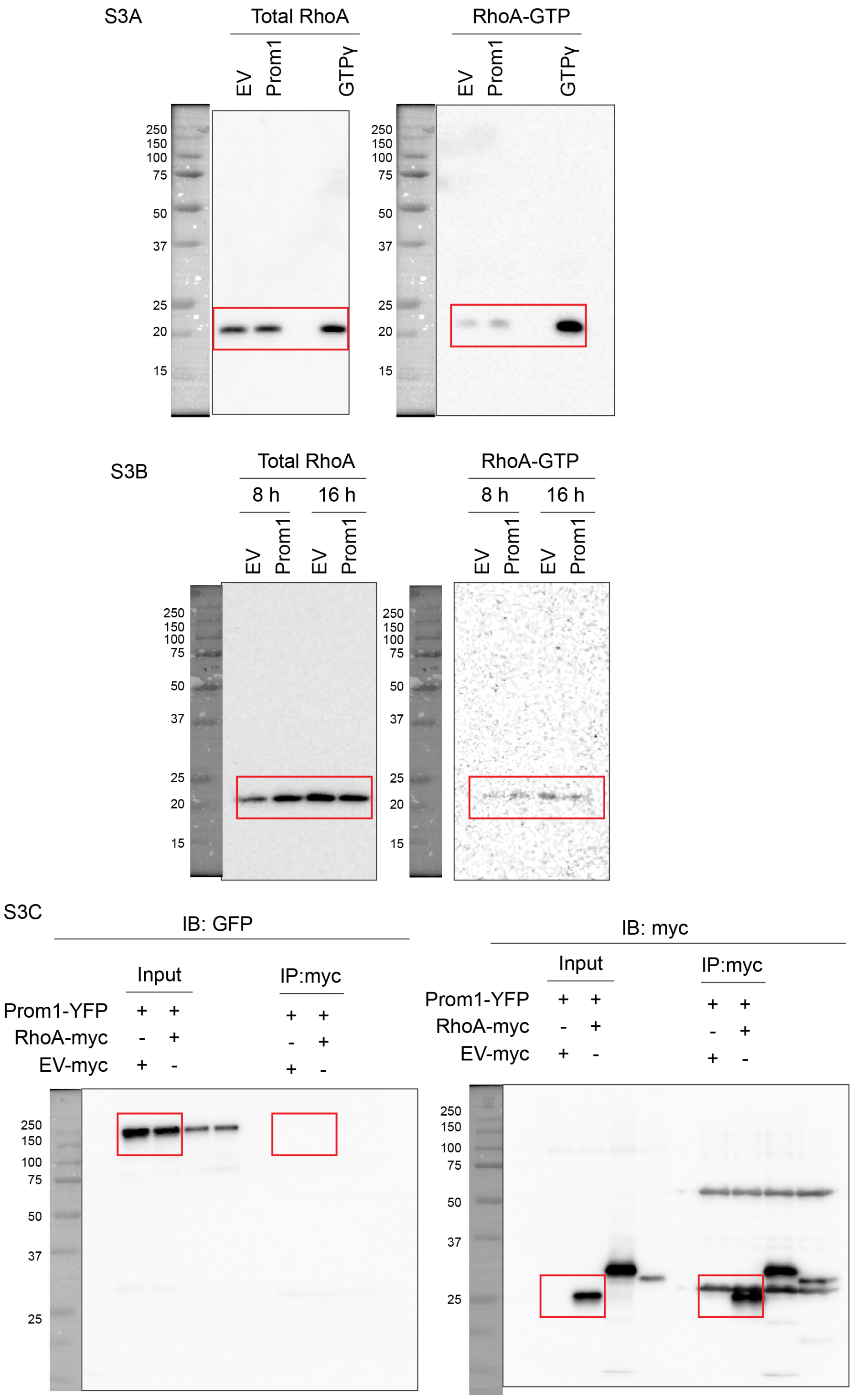
S3A
Total RhoA
RhoA-GTP
EV
Prom1
GTPγ
250
150
100
75
50
37
25
20
15
S3B
8 h
16 h
S3C
IB: GFP
IB: myc
Input
IP:myc
Prom1-YFP
RhoA-myc
EV-myc

Figure S8

S8B

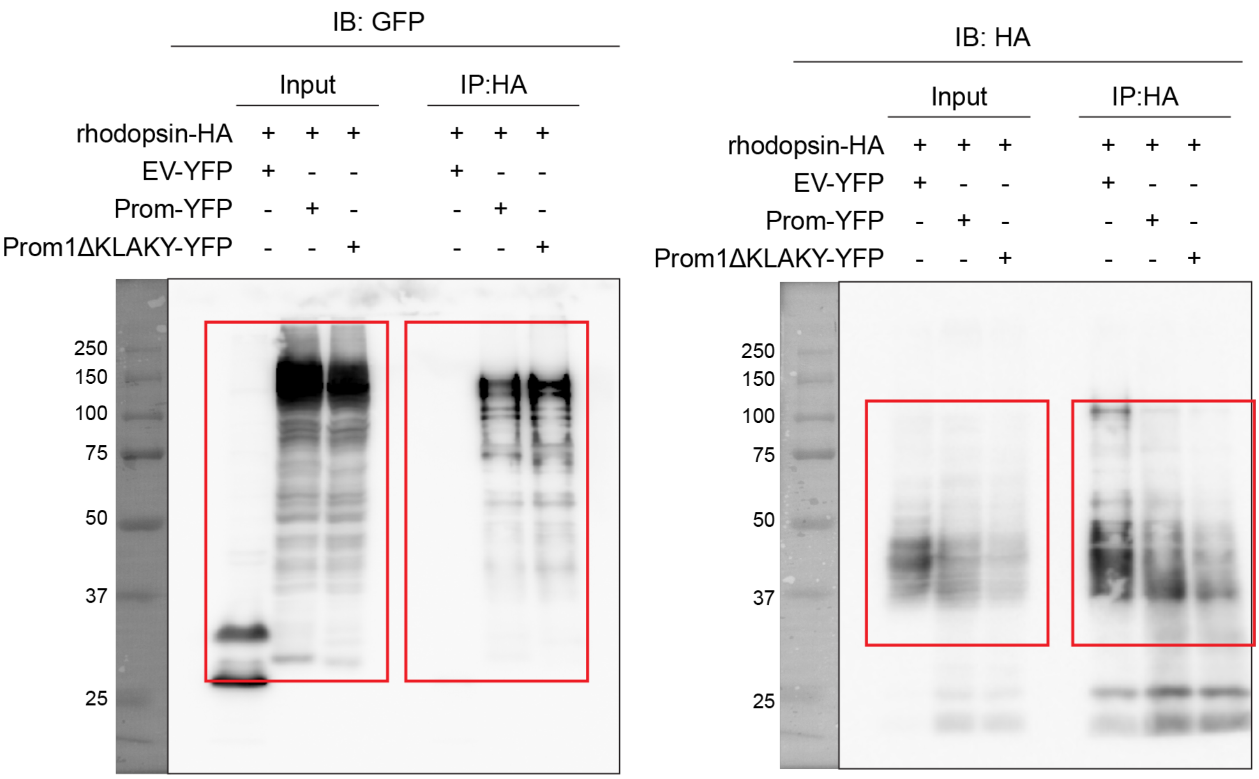

Supplementary Fig. 9 Original images of the western blots.

Supplementary Movies

(Movies have been submitted with separate files)

Supplementary Movie S1 The continuous pictures related to Fig. 1D were processed to the video.

Supplementary Movie S2 The continuous pictures related to Fig. 4F were processed to the video.

Supplementary Movie S3 The continuous MQAE images on wild-type (A) and *Prom1*KO cells (B), related to Fig. 5A were processed to the video.

Supplementary Movie S4 The continuous pictures on GFP (control GFP (A), Prom1FL (C) and Prom1ΔKLAKY (E)) and MQAE images (GFP (B), Prom1-FL (D) and Prom1-ΔKLAKY (F)), related to Fig. 5C were processed to the video.

Supplementary Movie S5 The continuous MQAE images with DMSO (A), Y-27632 (B) and C3 (C), related to Fig. 5D were processed to the video.
